# Leech therapy (*Hirudo medicinalis*) attenuates testicular damages induced by testicular ischemia/reperfusion in an animal model

**DOI:** 10.1186/s12917-021-02951-5

**Published:** 2021-07-27

**Authors:** Farshid Davoodi, Shayan Taheri, Abbas Raisi, Asghar Rajabzadeh, Amir Zakian, Mohammad Hassan Hablolvarid, Hassan Ahmadvand

**Affiliations:** 1 Department of Clinical Sciences, Faculty of Veterinary Medicine, Lorestan University, Khorramabad, Iran; 2 Razi Herbal Medicines Research Center, Lorestan University of Medical Sciences, Khorramabad, Iran; 3 Department of Anatomical Sciences, Faculty of Medicine, Lorestan University of Medical Sciences, Khorramabad, Iran; 4 Razi Vaccine and Serum Research Institute, Agriculture Research, Education and Extension Organization (AREEO), Karaj, Iran; 5 Medicinal Plants and Natural Products Research Center, Hamadan University of Medical Sciences, Hamadan, Iran

**Keywords:** Spermatic cord torsion, Testicular ischemia/reperfusion, Leech therapy, *Hirudo medicinalis*, Oxidative stress, Sperm parameters, Apoptosis

## Abstract

**Background:**

Testicular torsion/detorsion triggers tissue ischemia/reperfusion, leading to reactive oxygen species overgeneration and apoptosis. The saliva of leeches is full of anti-inflammatory, anticoagulants, antioxidants, and antimicrobial agents. Therefore, this study aimed to assess the protective mechanism of leech therapy on testicular ischemia/reperfusion damage.

**Methods:**

18 adult male rats were randomly divided into three groups: 1-Sham-operated group (SO). 2-Torsion/detorsion (T.D) group: two hours of testicular torsion with two hours of testicular detorsion was performed. 3-Torsion/detorsion + Leech therapy (TDL) group. Sperm parameters (motility, vitality, morphology, and concentration), oxidative stress biomarkers (MDA, CAT, GPx, and TAC), histopathological factors (Mean seminiferous tubular diameter, Germinal epithelial cell thickness, Testicular capsule thickness, Johnson’s score, and Cosentino’s score), and immunohistochemical markers for apoptosis detection (Bax, Bcl-2, and Caspase-3) were measured.

**Results:**

There was a significant difference for all sperm parameters in the T. D group compared to the sham group. Leech therapy significantly increased progressive motility and normal morphology and reduced non-progressive motility. In the TDL group, MDA concentration significantly reduced, and levels of GPx, TAC, and CAT remarkably increased. All evaluated histopathological parameters in the TDL group significantly increased compared to the T. D group except for the testicular capsule thickness. T. D notably increased the expression of Bax and Caspase-3, while the treatment group slowed the rate of apoptosis compared to the control group. Bcl-2 expression in the T. D group was significantly lower than that in the sham group. Leech therapy increased the Bcl-2 expression.

**Conclusion:**

Leech therapy attenuates damages to testicular tissue following torsion/detorsion due to its antioxidant, anti-inflammatory, and anti-apoptotic effects. Hence, it can be considered as an effective remedy for testicular ischemia/reperfusion.

**Graphical abstract:**

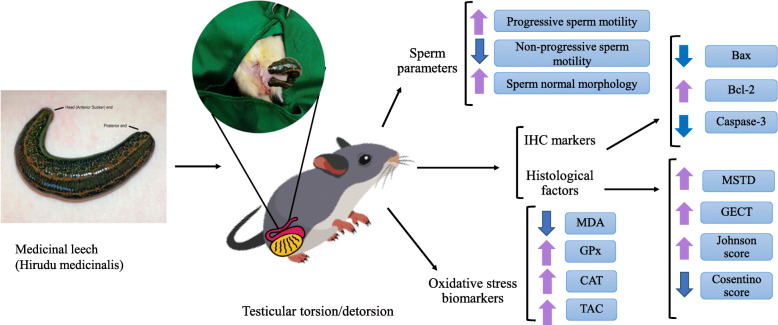

**Supplementary Information:**

The online version contains supplementary material available at 10.1186/s12917-021-02951-5.

## Background

Testicular torsion is known to be a critical medical emergency that requires quick intervention to preserve testicular function. One in 4000 men may experience this complication in their life [1]. Once this complication is diagnosed, surgical intervention should be performed as soon as possible to correct it [2]. The sooner this disorder is diagnosed and treated, the less testicular tissue remains in the ischemic state, and the injury to testicular tissue is reduced [3]. Ischemia/reperfusion (I/R) of testicular tissue occurring after torsion/detorsion is responsible for testicular damages and is known to be the main pathophysiology of this disorder [4]. Reactive oxygen species (ROS) overproduction, intracellular calcium overload, inflammatory cell reactions, coagulation and thrombosis, and germinal cell apoptosis happens following torsion/detorsion [4, 5]. Previous studies concerning testicular torsion revealed that several groups of drugs are effective in decreasing testicular injuries including antioxidants, calcium channel blockers, anti-inflammatory agents, vasodilator agents, anti-platelets, phytotherapeutics, and anti-thrombotic agents [6].

Testicular torsion happens in two manners of extravaginal and intravaginal. During the perinatal period, when the testicles descend into the scrotum, they may experience a rotation that occurs extravaginally. Intravaginal torsion most commonly occurs in puberty in which the spermatic cord and testis twist without torsion of tunica vaginalis [7]. Intravaginal torsion befalls due to an anatomical disorder which is named ‘bell clapper’. Tunica vaginalis has two layers, visceral and parietal. Normally, the parietal layer connects to the posterior part of the epididymis and testis. Once the parietal layer does not attach to the caudal epididymis and testis, the testicle is exposed to torsion [8]. There are two surgical methods for the treatment of this disorder, including orchiectomy and orchidopexy. A decision for the surgical method is made during the surgical procedure based on the testicular appearance. If the severity of ischemia is high, orchidectomy and otherwise orchidopexy is performed [9]. However, whether unilateral orchiectomy or orchidopexy is performed, it is suggested to perform an orchidopexy in the contralateral testis when bell clapper deformity is the reason of torsion [10].

Leech therapy is considered to be a kind of complementary or alternative medicine. The first usage of leeches in medicine was in Egypt, but in the seventeenth century, their use became widespread throughout the world [11]. More than 600 species of leeches have been recognized worldwide. However, only a few are used in medicine; the most famous of which is *Hirudo medicinalis* [12]*.* Food and Drug Administration (FDA) approbated the application of medicinal leech as a medical device in June 2004 [13]. The treatment properties of leeches are related to the site biting, blood suction, and most importantly, the injection of leech saliva into the site, which contains many bioactive substances [14]. Leech saliva of the *Hirudo medicinalis* consists of several substances that include Hirudin, Calin, Destabilase-lysozyme, Hyaluronidase, Bdellastasin (bdellin A), Tryptase inhibitor, Saratin, and γ-Glutamyl transpeptidase [15]. The most important substance in leech saliva is Hirudin which prevents blood clotting and acts as an anti-thrombotic agent [16]. Previous research has shown that leeches have analgesic and anti-inflammatory effects, blood flow enhancement, prevention of platelet aggregation, anticoagulant effects, and antimicrobial effects [11]. Anticancer effects, effects against cell degradation, application for skin flaps, wound healing improvement, usage in reconstructive surgeries, acute venous congestion recovery, and shielding effects against cerebral ischemia-reperfusion injury are all defined effects of leech therapy in the literature [11, 14, 17–19].

Medicinal leeches have been used for several purposes in previous studies, including improvement of venous congestion in rat epigastric flaps [20], glans penis congestion [21], replanted or revascularized digits [22], and various local flaps [23]. Moreover, leech therapy was employed in literature for plastic and reconstructive surgery, musculoskeletal diseases, migraine headaches, skin disorders, diabetic ulcers, priapism, cancer, wound healing, and osteoarthritis [24, 25]. Some previous studies have focused on leech application for reperfusion of ischemic tissues, such as the study conducted by Moosavian et al.; on cutaneous pedicle flaps exposed to extended ischemia, which revealed that flap survival rate of leech treated group was significantly higher than that in the control group [26]. Another study evaluated the protective effects of leech extract on cerebral ischemia/reperfusion and concluded that in the treatment group, apoptosis and necrosis of the nerve cells significantly diminished [17]. The antioxidant activity of the leech saliva extract was assessed by Ghawi et al. (2012). They employed DPPH free radical scavenging activity method and found that leech saliva extract revealed a potent antioxidant activity [27].

The well-known ingredient of leech saliva is hirudin. Prevention of blood coagulation is done by an anticoagulant compound in leech saliva, which is known as hirudin. It is a protein structure and possesses 65 amino acids with a high proportion of aspartic acid and glutamic acid. The anticoagulation effect is performed by binding with thrombin in the blood [28]. Previous studies have demonstrated the protective effects of heparin and anti-thrombotic drugs on testicular torsion/detorsion [5, 29]. The protective effects of the leech extract on damages caused by cerebral ischemia/reperfusion were demonstrated in previous studies [17]. However, the effects of leech therapy on testicular torsion/detorsion have not been investigated previously. Our aim in this investigation was to examine whether leech therapy can protect testicular tissue from ischemia/reperfusion damage in rats.

## Methods

### Leech preparation

Twenty-five medical leeches of *Hirudo medicinalis* species were obtained from Karaj Biological Research Farm, Karaj, Iran. Leeches were stored in large jars containing chlorine-free water in a cool, semi-bright environment for one week. The jars were covered with a large net to prevent the leeches from coming out of the container. One-third of the container water was replaced with fresh, chlorine-free water every three to 4 days. Leeches were then used on the day of the experiment.

### Animals and experimental groups

Eighteen adult male rats were provided by the Razi Herbal Medicines Research Center of Lorestan University of Medical Sciences. The average body weight of animals was 230 ± 30 g (10–11 weeks old). The animals were kept in standard polypropylene cages with a temperature of 24.5 ± 0.5 °C and humidity of almost 50% with a 12 h light/12 h dark cycle. Rats were provided with a standard rodent diet (Behparvarco® Lab Animals Diet, Tehran, Iran) and drinking water ad libitum. Prior to commencing the study, ethical clearance was sought from the Animal Ethics Committee (LU. ACRA.2018.13, Lorestan University, Faculty of veterinary medicine). All employed methods in this study were carried out in accordance with relevant guidelines of Lorestan University Animal Ethics Committee. This work has been reported in accordance with the ARRIVE guidelines (Animals in Research: Reporting In Vivo Experiments) [30].

The animals (*n* = 18) were randomly separated and split into three groups of six as follows:

1. Sham-operated (SO) group: Left testicle was exposed by an incision in the scrotum, pulled out, and was back to its normal position without any torsion induction (*n* = 6).
2. Torsion/detorsion (T.D) group: Testicular torsion was surgically induced and maintained for 2 h. subsequently, detorsion was performed and remained for 2 h (*n* = 6).
3. Torsion/detorsion + Leech therapy (TDL) group: Torsion of left testis was performed, and 30 min before detorsion, leech therapy was done for 7 min on the left scrotum (*n =* 6). The leech was placed on the left scrotum, and the time was recorded from when it started biting and blood-sucking until seven minutes. Thereafter, salt was utilized to detach the leeches from the scrotum. Based on previous studies, leeches can suck 5–15 ml of blood during 30 min [31, 32]. As there was no previous study on leech therapy on testicular torsion/detorsion, the dose was calculated as follows:

The normal blood volume in rats is 10% of their total body weight, and allowed blood volume for sampling without any complications is 10% of the total blood volume [33]. Each leech can ingest 5–15 ml of blood for 30 min based on its body size [32]. Therefore, in the present study, according to rats’ weights (230 ± 20 g), the total volume of blood was 23–25 ml, and we were allowed to take blood samples up to 2.3–2.5 ml. A seven min leech therapy was performed, which did not cause any related complications.

### Surgical induction of torsion

The surgical procedures for torsion induction were performed in 4 stages of anesthesia, torsion induction, detorsion, and orchidectomy based on previous studies [34, 35] as follows:

1. Intraperitoneal (i.p) injection of xylazine and ketamine was used to induce anesthesia (5 mg kg^− 1^, 60 mg kg-1, Alfasan, Woerden, The Netherlands). Scrotal hairs were clipped, and a povidone-iodine 10% solution was employed for disinfection of the surgical site. Subsequently, three drapes were placed in a clockwise direction using towel clamps.
2. An incision was made on the midline of the scrotum using a scalpel blade No. 15. Then covering layers of the left testis, including tunica vaginalis, were incised, and the left testis was exposed. Testicular ischemia was performed by rotating the left testis 720 degrees clockwise, and tunica albuginea was sutured to the scrotum in order to stabilize the testicle in this state.
3. After 2 h of testicular ischemia, reperfusion was performed by rotating the left testis 720 degrees counterclockwise. Afterward, the reperfused testis was placed back into the scrotum, and the scrotum was stitched.
4. Two hours following detorsion, all animals were euthanized using an overdose of thiopental sodium (250 mg/kg, i.p. Exipental, Exir, Borujerd, Iran), and 2 ml cardiac blood samples were collected, and orchidectomy was done. Blood samples were centrifuged at 3000 RPM for 8 min to separate the plasma. Subsequently, plasma was removed using an adjustable micropipette (Brand, Germany) and added to 1.5 ml microtubes and frozen at − 80 °C. Left epididymis was utilized for sperm quality assessment. Following orchidectomy, by a small incision, a sample was taken from the left testis for the oxidative stress evaluation of the testis tissue (MDA, GPx, CAT, and TAC) and sent to the biochemistry lab in the containers containing cold packs and the rest was fixed in the 10% formalin buffer solution. No deaths or major complications were detected during the experiments.

An additional MP4 file shows the surgical procedure in more detail (see Additional file 1).


**Additional file 1.** This file (MP4) shows the surgical procedure used to induce testicular torsion in rats. 

### Sperm quality assessment

#### Sample collection

To collect mature sperm cells from epididymis, the method described by Varisli et al. was utilized [36]. 5 ml of RPMI 1640 medium (Sigma-Aldrich, Munich, Germany) was poured into a small petri dish. Subsequently, the posterior part of the epididymis was cut using small sharp scissors and inserted in the petri dish containing RPMI medium. Several incisions were made in the epididymis to facilitate sperm suspension in medium culture. Eventually, the sample was kept in a 37 °C incubator for 10 min to help sperm drainage.

#### Measured parameters

**Fig. 1:**
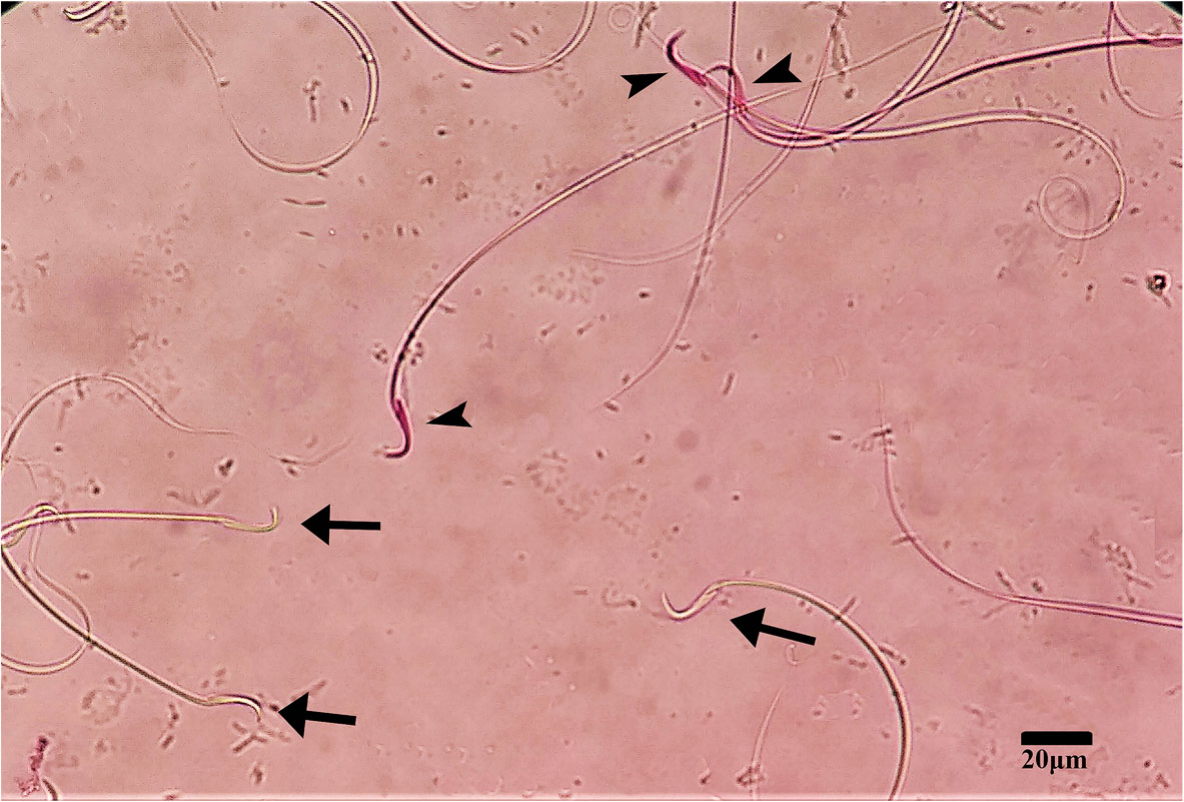
Sperm vitality assessment using the eosin staining method. Arrowheads show dead spermatozoa and arrows show live spermatozoa


Sperm quality evaluation was done based on the protocols defined by World Health Organization (WHO) [37]. According to WHO, initially, sperm motility should be assessed. A phase-contrast microscope (Olympus IMT-2, Japan) with X 400 magnification was employed to examine sperm motility in three various categories of progressive motility (PR), non-progressive motility (NP), and immotility (IM). Three Supplementary files show sperm motility in different groups of the study (Additional files 2, 3, and 4). Subsequently, the viability of sperm cells was assessed. Staining with eosin was employed for viability examination. The basis of this method is based on the penetration of eosin into sperm cells [37, 38]. This means that the eosin penetrates dead cells, and they are observed under the microscope in pink while living sperms are resistant to stain penetration and remain colorless (Fig. 1). A light microscope (CX21, Olympus, Japan) with X 100 magnification was utilized to evaluate sperm vitality. To determine sperm concentration, initially, a standard fixative was made. The fixative solution was made from combining 0.5 g of NaHCO3 and 1 ml of 35% formalin solution in 100 ml of pure water according to the WHO laboratory manual for the examination and processing of spermatozoa [37]. The purpose of making a fixative solution and mixing it with spermatozoa is to kill sperm cells to stop their movement and ease of counting. Based on WHO, different dilution rates can be used to mix the fixative solution with spermatozoa samples,including 1:20, 1:5, and 1:2 [37]. In the present study, we utilized a 1:20 dilution rate, and 50 μL of the sample was mixed with 950 μL of the prepared fixative solution. Then, 10 μl of the diluted sample was loaded on the hemocytometer chamber (Paul Marienfeld GmbH & Co. KG, Lauda-Königshofen, Germany), and sperm cells were counted under a phase-contrast microscope (Olympus IMT-2, Japan). The ultimate concentration of spermatozoa was presented as sperm per ml. Eventually, the morphology of spermatozoa was assessed using the Papanicolaou staining method. Hematoxylin Papanicolaou (Sigma-Aldrich, Munich, Germany) solution was employed to stain sperm cells, and morphological examination was performed under a light microscope. Based on WHO, sperm cells with coiled tails, bent tails, short tails, detached heads with a short tail, cytoplasmic droplets, and elongated heads were considered abnormal morphologically. All methods regarding sperm parameters evaluation are available in detail in our previous studies [2, 29].

**Additional file 2.** This file (MP4) shows sperm motility in different classifications of PR, NP, and IM in the sham group. 


**Additional file 3.** This file (MP4) shows sperm motility in different classifications of PR, NP, and IM in the T. D group. 


**Additional file 4.** This file (MP4) shows sperm motility in different classifications of PR, NP, and IM in the TDL group. 

### Oxidative damage evaluation

Oxidative stress intensity was evaluated in testicular tissue and plasma samples. The colorimetrical commercial biochemical kits of Asan (Asan, Khorramabad, Iran) were employed to assess the amount of total protein (TP), glutathione peroxidase (GPx), catalase activity (CAT), total antioxidant capacity (TAC), and malondialdehyde (MDA). All methods to examine oxidative damage were available in the kit protocol guide and were followed step by step.

Thiobarbituric acid (TBA) assay was utilized to measure MDA level in the samples based on the method described by Ahmadvand et al. (2014) [39]. Plasma and testicular tissue samples (100 μl) were combined with TBA solution (1500 μl) and TCA solution (100 μl) and incubated in a water bath (KTG water bath, Tehran, Iran) with a temperature of 95 °C for 30 min. Subsequently, 15 min centrifuge at 1000 rpm was performed. Afterward, the supernatant was removed, and the absorbance of the sample was read at 535 nm using a spectrophotometer (JENWAY 6715 UV/Vis Spectrophotometer, Staffordshire, UK). The final concentration of MDA was expressed as μmol/mg-pr.

Catalase activity was evaluated according to the method of Sinha with slight modification [40]. 50 μl of the sample was added to 1000 μl of potassium phosphate buffer (pH = 7). Subsequently, 50 μl of hydrogen peroxide (H_2_O_2_) with 30 mmol/L concentration was added, and the concentration of the catalase was read at 240 nm using (JENWAY 6715 UV/Vis Spectrophotometer, Staffordshire, UK). Sample catalase concentration was reported as units/mg-pr.

Glutathione peroxidase (GPx) level was measured according to the method of Ahmadvand et al. [39]. The kit protocol for GPx was as follows, 200 μl of Tris HCl buffer solution, 100 μl of sodium azide, 200 μl of glutathione, 100 μl of hydrogen peroxide (H_2_O_2_), and 200 μl of samples were all combined in a tube and incubated in a water bath (KTG water bath, Tehran, Iran) at 37 °C for 10 min. Then, 400 μl of 10% TCA solution was added, and the final solution was centrifuged at 3000 rpm for 3 min. Subsequently, 25 μl of the supernatant was removed and added to 140 μl of Tris EDTA solution. Afterward, 30 μl of the DTNB solution was added to the mixture. Ultimately, 30 min of incubation at room temperature was performed, and absorption of the solution was read at 450 nm using an ELISA reader (Awareness Technology Stat Fax 3200 Microplate Reader, Ramsey, Minnesota). GPx concentration was reported as units/mg-pr.

Assan kit protocol for total antioxidant capacity (TAC) was set based on the method described by Koracevic et al. (2001) [41]. Initially, 75 μl of the sample was mixed with 500 μl of reagent solution 1 (H_2_SO_4_). Then, 20 μl of reagent solution 2 (H_2_SO_4_ + CuSO_4_ + o-Dianisidine) was added to the prepared solution. Eventually, the absorption of the solution was examined at 630 nm by Microplate Reader, and the TAC level was expressed as nmol Trolox/mg-pr.

### Histopathological examinations

The tissue processing procedure was performed based on previously published studies [42, 43]. Samples that were taken from testicular tissue after orchidectomy surgery and fixed in a 10% formalin buffer solution were utilized for pathological evaluations. Initially, samples were removed from the fixative solution, and dehydration was carried out at ascending levels of ethyl alcohol. Xylene was then utilized to clear tissue samples, and embedding in paraffin was performed. Using a rotary microtome (Leica, rm2235, Nussloch, Germany), 4–5 μm thick sections were prepared from blocks. Subsequently, the sectioned samples were mounted on slides and stained using the hematoxylin and eosin (H&E) staining method. Afterward, provided slides were assessed under a light microscope (CX21, Olympus, Japan) by an expert pathologist who was fully blinded to experiments.

Histopathological parameters due to previous investigations [44], including mean seminiferous tubular diameter (MSTD), germinal epithelial cell thickness (GECT) according to μm thickness, and the number of cell layers, and testicular capsule thickness (TCT), were evaluated using a microscope eyepiece with reticle. A quantitative approach of Johnson was employed in 10 levels to score spermatogenesis in all experimental groups of the study based on the cells involved in spermatogenesis, including spermatozoa, late spermatids, early spermatids, spermatocytes, and spermatogonia [45]. Cosentino’s method was employed to assess testicular injury and necrosis. This method includes 4 degrees categorized according to necrosis of testicular tissue, organization of germ cells, and hemorrhage [46]. To control for bias, measurements were carried out by two expert pathologists.

### Immunohistochemical evaluations

Prepared tissue sections were dewaxed and hydrated using descending levels of ethanol. Subsequently, an immunohistochemistry staining protocol was performed based on previous studies [47]. Herein, we employed IHC markers including Bax, Bcl2, and caspase-3 to detect apoptosis in testicular tissue and germinal cells using Elabscience primary and secondary antibodies (Elabscience, Wuhan, China).

### Statistical analysis

To examine the impact of leech therapy on oxidative stress indicators, sperm parameters, and histopathological changes, comparative analyses were performed by MedCalc software (Ver. 19.2, Ostend, Belgium) and Analyse-it software (Ver. 5.51.1, Leeds, UK). Significance levels were set at the ≤0.05 level. For the sham-operated, T. D, and TDL groups, descriptive statistics were determined on each variable separately. The Kolmogorov-Smirnov single sample method was utilized to evaluate the distribution of data. Normal distribution data were expressed as mean ± standard deviations, and data with abnormal distribution were described as median and interquartile scales. One-way ANOVA with Tukey-Kramer post hoc was used for analyzing data with normal distribution, and groups with abnormally distributed data were examined by the non-parametric Kruskal-Wallis test.

## Results

### Sperm quality assessment findings

Table 1 presents sperm parameters in different groups. The difference between the sham-operated group and the T. D group was significant for all measured parameters (*P* < 0.05). In the TDL group, Leech therapy significantly increased the progressive motility and reduced the non-progressive motility of sperm cells (*P* < 0.05). No significant difference was observed between the TDL group and T. D group for sperm vitality and sperm concentration (*P* > 0.05). Table 2 provides the results obtained from the preliminary analysis of sperm morphology in the studied groups. Induction of testicular torsion/detorsion significantly reduced the percentage of normal spermatozoa and increased the percentage of coiled tail sperm cells compared to the sham group (*P* < 0.05). Leech therapy noticeably enhanced the percentage of normal sperm cells than that in the T. D group (*P* < 0.05). No significant difference was observed for other measured values between the TDL and T. D groups (*P* > 0.05).

**Table 1:** Sperm parameters examination in different groups of the study. Data with normal distribution are displayed as Mean ± SD; Non-normal distribution data are expressed as median and interquartile range

Sperm parameters	Sperm motility (%)			Sperm vitality (%)	Sperm concentration × 10^6^ sperm/ml
Groups	Progressive	Non-Progressive	Immotile		
Sham	62.89 ± 1.82	7.30(5.36,10.16)	29.58 ± 1.22	65.62 ± 1.80	63.81(54.32,68.30)
T. D	37.05 ± 1.74^a^	21.25(18.99,25.74)^a^	41.33 ± 2.16^a^	47.12 ± 2.65^a^	50.48(32.96,74.37)^a^
TDL	55.25 ± 2.60^b^	8.08(4.80,15.20)^b^	37.22 ± 1.45	58.80 ± 2.87	57.62(53.50,66.50)

^a^*p* < 0.05 compared with the sham group

^b^*p* < 0.05 compared with the T-D group

**Table 2:** Morphology of cauda epididymal spermatozoa. Data with normal distribution are displayed as Mean ± SD; Non-normal distribution data are expressed as median and interquartile range

Groups	Normal morphology (%)	Bent tail (%)	Coiled tail (%)	Distal cytoplasmic droplet (%)	Short tail (%)	Abnormal head (%)
Sham	84.23 ± 2.24	12.18 ± 2.32	2.31(1.20,3.00)	0.32 ± 0.53	0 ± 0.00	1.41 ± 1.36
T. D	70.34 ± 1.19^a^	20.35 ± 2.28	7.98(3.84,4.27)^a^	1.24 ± 0.79	0.41 ± 0.25	0 ± 0.00
TDL	79.66 ± 3.51^b^	13.08 ± 3.68	5.36(0.00–10.13)^a^	0 ± 0.00	0.16 ± 0.17	0.50 ± 0.38

^a^*p* < 0.05 compared with the sham group

^b^*p* < 0.05 compared with the T. D group

### Oxidative damage

**Fig. 2:**
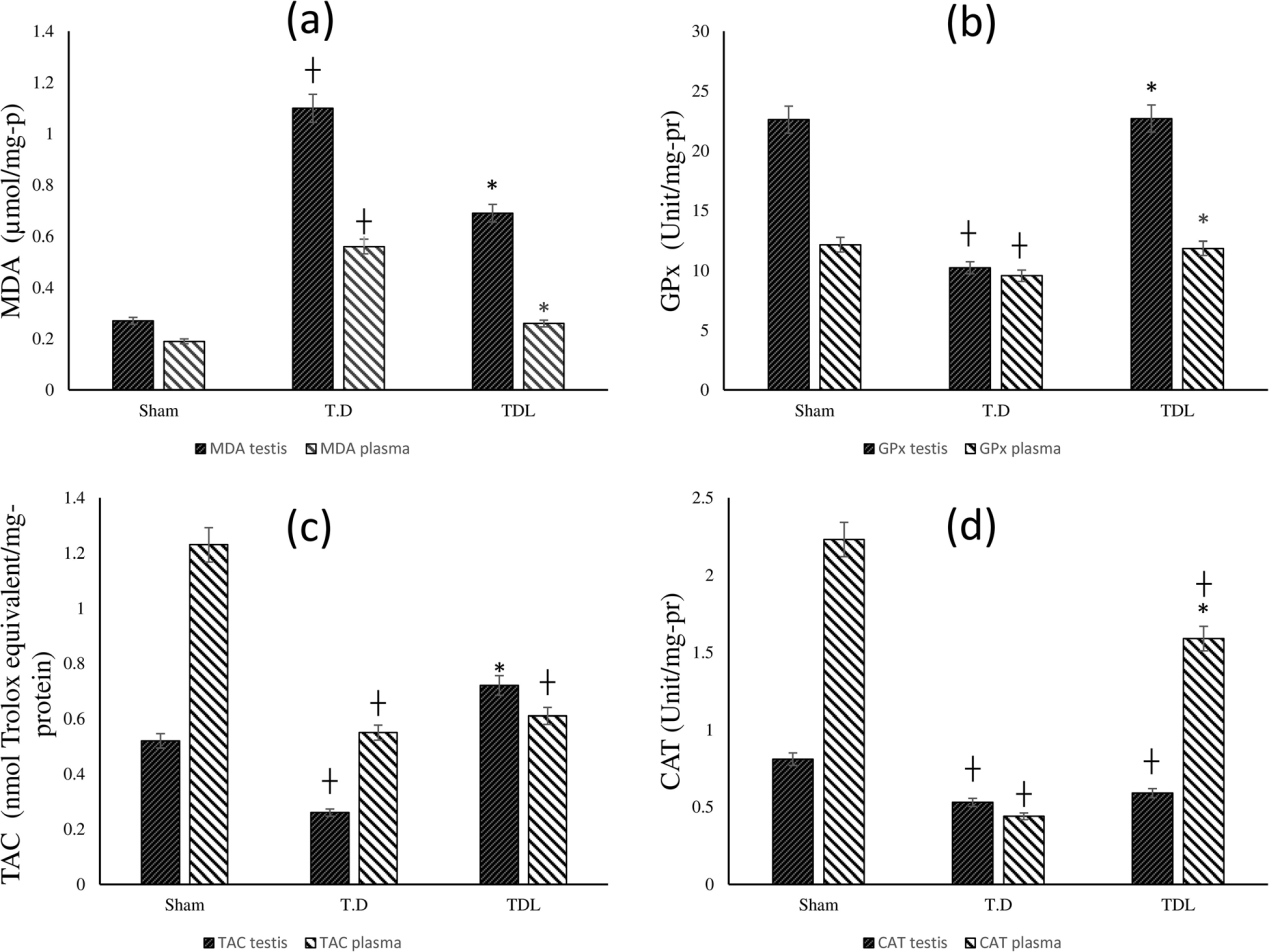
Oxidative damage evaluation in testicular tissue and plasma. **a** MDA concentration; **b** level of GPx; **c** TAC level; **d** level of CAT

The results of the oxidative damage evaluation are presented in Fig. 2. As shown in Fig. 2a in the T. D group, testicular and plasma concentrations of MDA were remarkably higher than that in the sham group. MDA concentration of testicular tissue and plasma was significantly reduced in the TDL group compared to the T. D group (*P* < 0.05). Figure 2b presents the level of GPx in all groups of the study. Testicular torsion/detorsion significantly reduced the testicular and plasma levels of GPx in comparison to the sham group (*P <* 0.05). Treatment with leeches significantly enhanced the levels of GPx in comparison to the T. D group in both testicular and plasma samples (*P* < 0.05). The total antioxidant capacity (TAC) of testicular and plasma tissues is represented in Fig. 2c. As can be seen from the graph, the difference between the sham group and the T. D group was significant (*P* < 0.05). TAC level of testis tissue in the TDL group was significantly higher than that in the T. D group (*P <* 0.05). Leech therapy increased the plasma level of TAC compared to the T. D group, but the difference was not significant (*P* > 0.05). Figure 2d shows the experimental data on catalase enzyme activity. Based on this graph, testicular torsion/detorsion significantly reduced catalase activity in the T. D group for both measured samples compared to the sham group (*P <* 0.05). The Plasma CAT level was remarkably higher in the TDL group than that in the T. D group (*P <* 0.05). There was no significant difference in testicular CAT levels between the TDL group and the T. D group (*P >* 0.05).

### Histopathological results

**Fig. 3:**
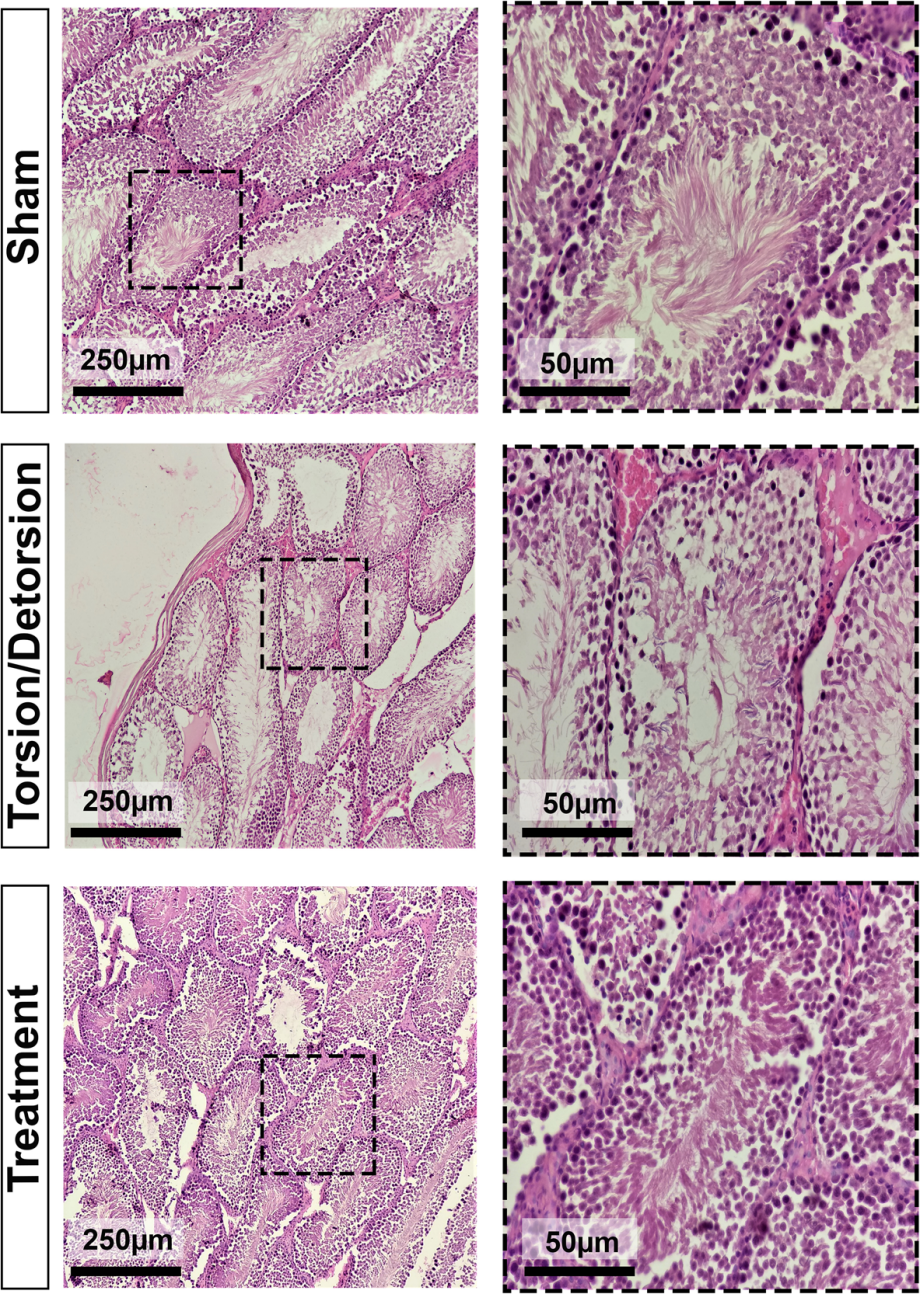
Hematoxylin and Eosin staining method for evaluation of tissue damages in different experimental study groups. In the sham group, the seminiferous tubules were healthy. In the T. D group hyperemia, intertubular hemorrhage, vacuolation, and desquamation of cells into seminiferous tubules were detected. In the treatment group, intertubular hemorrhage and mild vacuolation in some tubules were observed

**Fig. 4:**
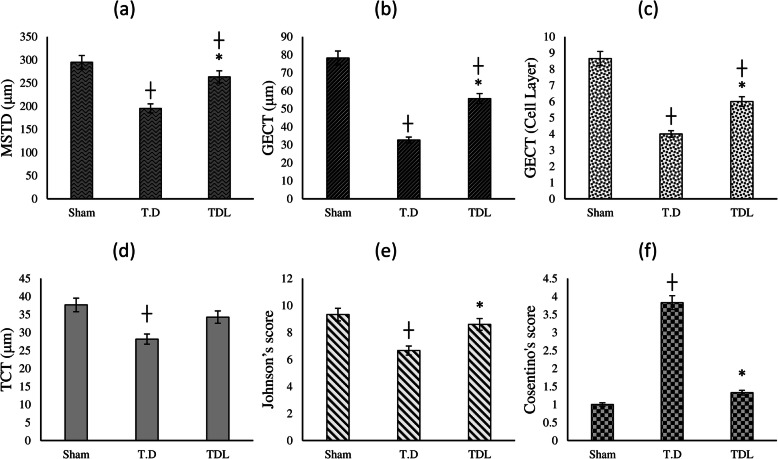
histopathological parameters. **a** mean seminiferous tubular diameter (MSTD); **b** germinal epithelial cell thickness (GECT); **c** GECT based on cell layer; **d** testicular capsule thickness (TCT); **e** Johnson’s score; **f** Cosentino’s score

Histopathological slides are presented in Fig. 3. In the sham group, the seminiferous tubules were healthy. Testicular I/R in the T. D group triggered considerable damage to the germinal cells, coagulative necrosis, intertubular hemorrhage, hyperemia, vacuolation, and desquamation of cells into seminiferous tubules were detected. Leech therapy in the TDL group improved the testicular injuries due to the ischemia/reperfusion, and just a slight intertubular hemorrhage and mild vacuolation in some tubules were observed. The results obtained from the preliminary analysis of histopathologic parameters are summarized in Fig. 4. Based on Fig. 4, the difference between the T. D group and the sham group for all measured values was significant (*p* < 0.05). As shown in Fig. 4a, MSTD in the TDL group was notably higher than that in the T. D group (*p <* 0.05). According to Fig. 4b, GECT based on the μm significantly increased in the TDL group compared to the T. D group (*p <* 0.05). Figure 4c indicates that GECT (cell layer) was remarkably higher in the TDL group in comparison to the T. D group (*p <* 0.05). Figure 4d presents the amount of TCT in different groups. As it is apparent from the graph, no significant difference was observed between the TDL group and the T. D group (*P* > 0.05). Figure 4e& f shows Johnson’s score and Cosentino’s score, respectively. According to graphs, Johnson’s score remarkably increased in the TDL group in comparison to the T. D group (*p <* 0.05). Leech therapy notably diminished Cosentino’s score compared to the T. D group (*p <* 0.05).

### Immunohistochemical results

**Fig. 5:**
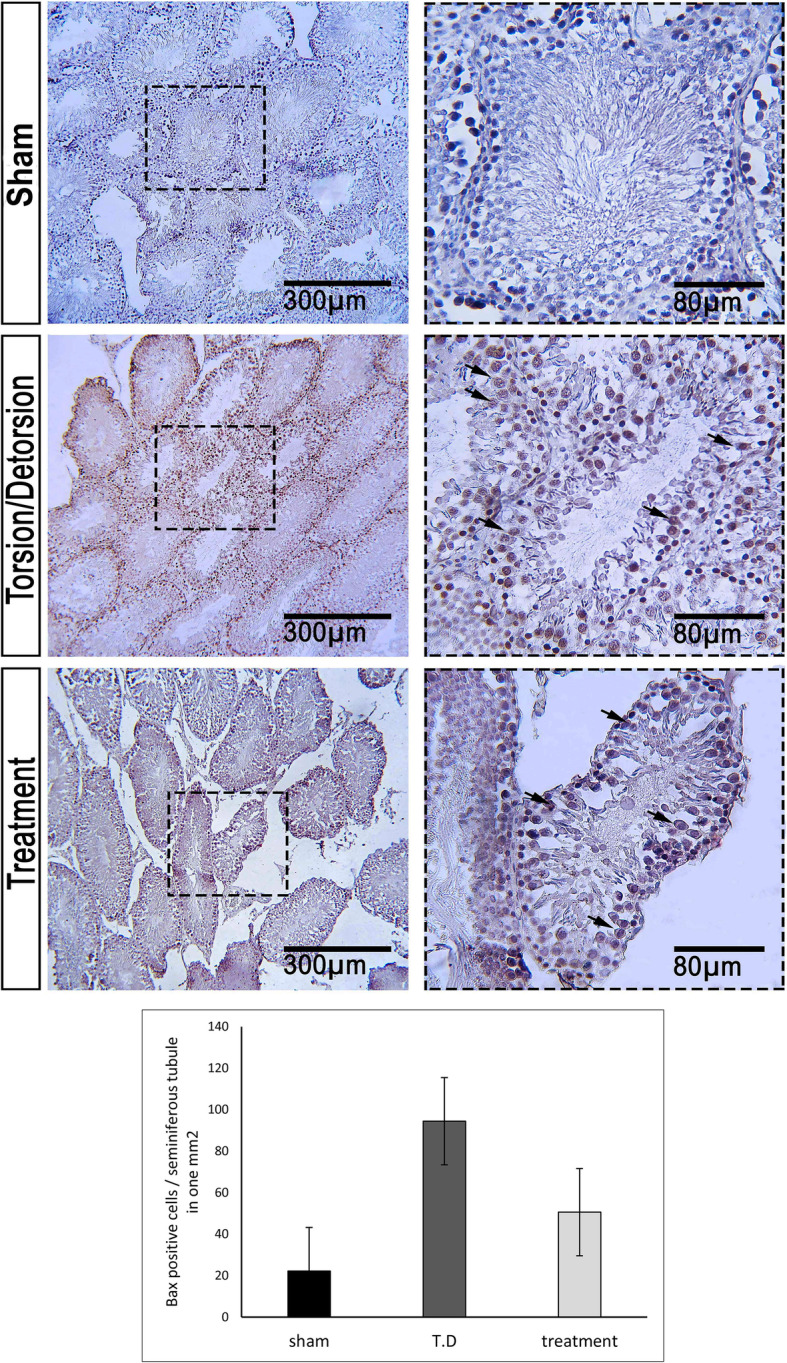
Expression of BAX in different groups. In the sham group, a low percentage of cells were positive for BAX. In the T. D group, a high percentage of cells were positive for BAX. In the treatment group, BAX expression was reduced

**Fig. 6:**
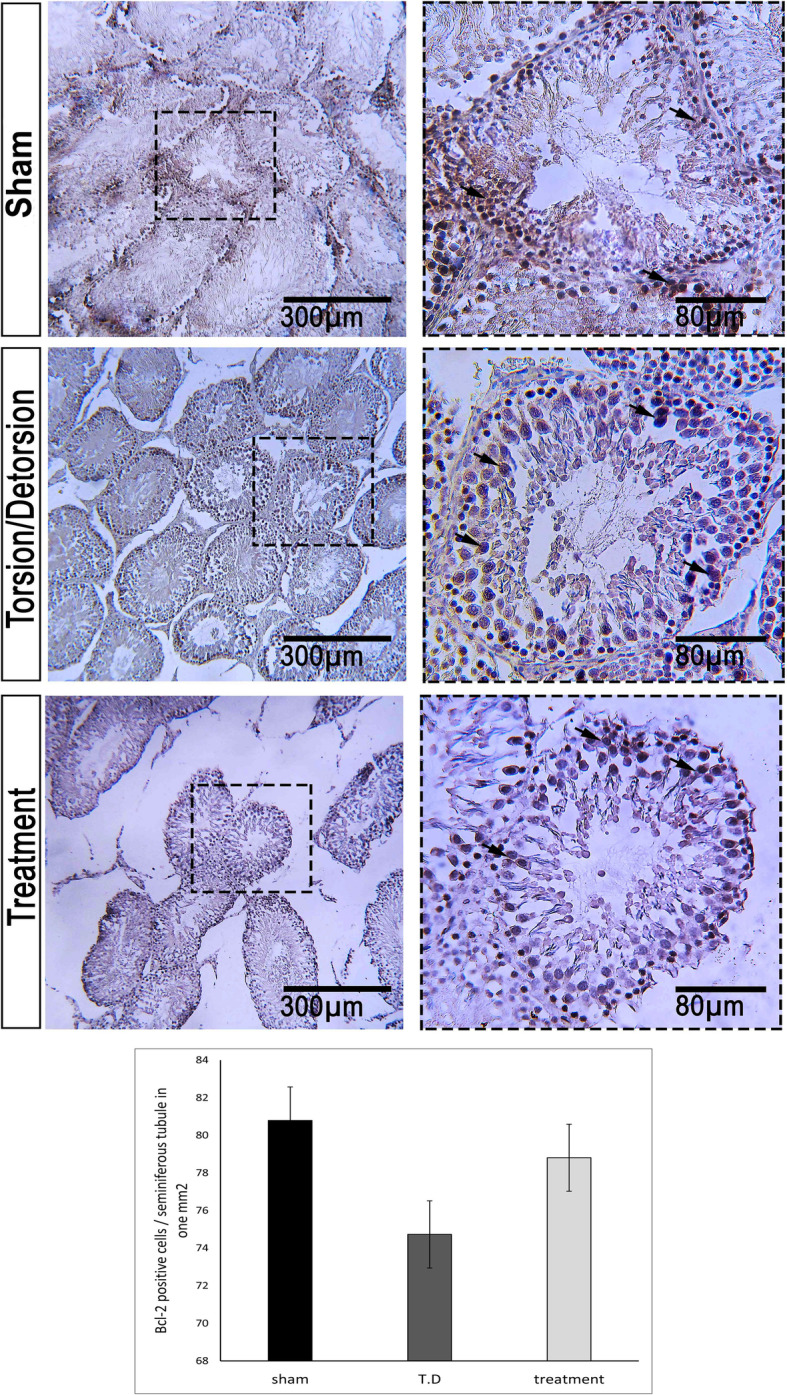
Bcl-2 expression in various groups of the study. In the sham group, Bcl-2 was extensively expressed. T. D revealed a mild Bcl-2 expression. In the treatment group, Bcl-2 was highly expressed

**Fig. 7:**
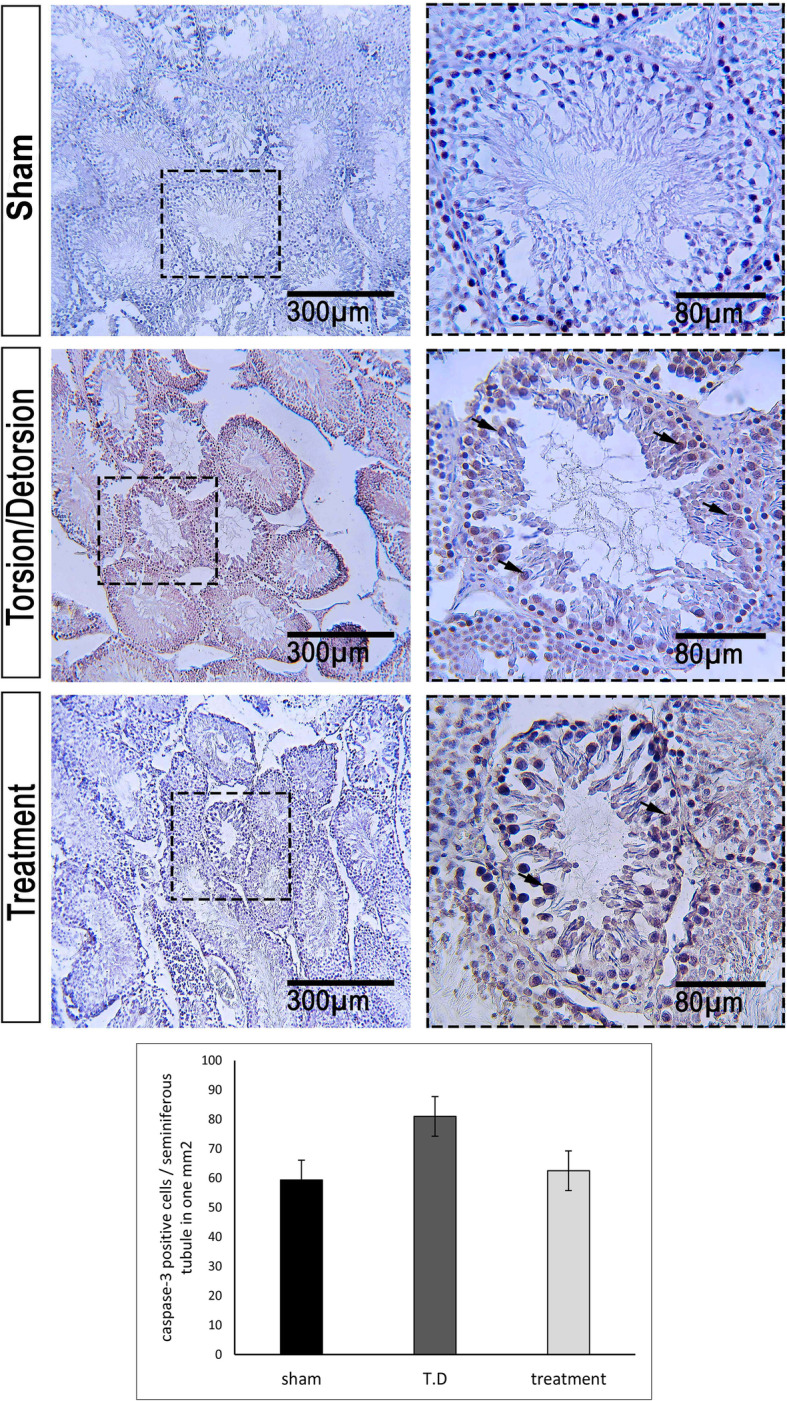
Caspase-3 expression in different groups. Low cells were positive for Caspase-3 in the sham group and Caspase-3 was highly expressed in the T. D group. The treatment group indicated lower positive cells for Caspase-3

Percentage of Bax positive cells per one seminiferous tubule in one mm^2^ was 22.16 ± 3.89, 94.41 ± 3.82, and 50.56 ± 10.93 in the sham, T. D, and TDL groups, respectively (Fig. 5). Bcl-2 was expressed in all groups. The percentage of Bcl-2 expression in the sham group was 80.79 ± 6.31 and in the T. D group was 74.83 ± 8.5. Leech therapy increased the Bcl-2 expression (78.81 ± 13.52) (Fig. 6). Expression of Caspase-3 was observed in all groups (Fig. 7). The percent of Caspase-3 expression was 59.33 ± 14.62, 80.96 ± 8.31, and 63.38 ± 14.63 in the sham, T. D, and TDL groups, respectively.

## Discussion

Torsion of the spermatic cord is known to be one of the prevalent urological emergencies which trigger testicular ischemia, apoptosis of germinal cells, and necrosis of the affected testis if not treated [48]. Owing to the terminal blood supply of the testicular tissue and inelastic covering layer of testes, ischemia induces abundance damages to the affected testis [49]. Inflammatory cell reactions and germinal cell apoptosis are considered the most important pathological mechanisms involved in testicular I/R [48]. Furthermore, the uncontrollable production of reactive oxygen species (ROS) is another reason for cellular damages following ischemia, and cell ingredients including nucleic acid, proteins, and lipids are vulnerable to ROS [50–52].. Blood flow correction and testicular reperfusion are the main aims of the treatment following testicular torsion [53]. Several types of research have been conducted to find the best medication to reduce damages due to testicular torsion detorsion, and the ultimate purpose of them is to translate the findings of animal studies to clinical trials and, subsequently clinical practice.

As proven in the previous studies, the most important agent of leech saliva involved in the coagulation process is hirudin which is comparable with heparin [19]. Qiu et al. (2018) have investigated the antiplatelet aggregation effects of leech and found that prevention of ADP-induced platelet aggregation, reducing the amount of Ca2+ and TXA2, the increment of PGI2 are several mechanisms involved in antiplatelet aggregation [54]. In research conducted by Mousavi et al. (2016), the effects of Hirudo therapy and heparin treatment in hindlimb acute venous congestion of rat were evaluated and concluded that leech therapy remarkably reduced edema, blood levels of creatine phosphokinase, tissue damage, and overall leech therapy was more effective in decreasing the cellular damages triggered by acute congestion [19].

Our findings revealed that testicular ischemia/reperfusion caused oxidative damage by increasing MDA levels and reducing GPx, TAC, and CAT both in the plasma and testicular tissue samples compared to the sham group. Leech therapy reduced oxidative damage by decreasing MDA levels and increasing GPx, TAC, and CAT levels compared to the T. D group. These results match those observed in earlier studies. In previous research, protective effects of leech micro powder on cerebral ischemia/reperfusion were examined, and it was found that activity of SOD increased in the cerebral tissue and serum, and the amount of MDA and NO diminished. Furthermore, the leech treated group decreased the apoptosis of cerebral cells [17]. In a study on the effects of heparin in ischemia/reperfusion injury of testis in rats conducted by Mertoglu et al. (2015), it was found that treatment with heparin notably diminished levels of PC and MDA in the testicular tissue, but did not significantly change the amount of NO, SOD and GPx [55]. Our previous research on the testicular torsion/detorsion and pre-treatment with heparin revealed that heparin significantly reduced MDA and increased CAT and GPx enzymes [29].

Evaluation of sperm parameters in the present study indicated that testicular torsion/detorsion reduced progressive motility and increased non-progressive and immotile sperm cells compared to the sham group. Moreover, sperm vitality and concentration were significantly reduced. In the TDL group, progressive motility was increased, and non-progressive spermatozoa reduced compared to the T. D group. Sperm morphology assessment revealed that following testicular torsion, sperm cells with normal morphology were reduced, and coiled tail spermatozoa was increased in comparison to the sham group. Leech therapy significantly increased the normal morphology of the spermatozoa. Perhaps, the antioxidant and anti-coagulant components of the leech saliva prevent damages due to ROS increment. No previous research has examined the effects of leech therapy on sperm parameters. However, in our previous research on the testicular torsion/detorsion and pre-treatment with heparin as an anticoagulant agent, it was demonstrated that heparin significantly increased sperm motility, sperm concentration, and normal morphology [29].

Present research indicated that testicular appearance following leech therapy in the TDL group was similar to the sham group, and less evidence of ischemia was observed in testicular tissue. Additionally, histopathological evaluation of the TDL group showed less tissue necrosis and damages following reperfusion. These results are consistent with those of other studies and suggest that leech therapy increases the salvage rate of the tissues. Lee et al. (2018) examined Hirudo therapy for digit revascularization and concluded that leech therapy over 4 days triggered more survival rates of digits [56]. Moosavian et al. (2014) investigated leech therapy’s effects on cutaneous pedicle flaps and found that leeching significantly increased flap survival [19]. Chepeha et al. researched the effects of leech therapy on venous obstruction following reperfused tissue transfer and found that leech therapy significantly increased the salvage rate of transferred tissue [57].

Histopathological findings indicated that testicular I/R remarkably damaged testicular tissue and hyperemia, intertubular hemorrhage, vacuolation, and desquamation of cells into seminiferous tubules were found in the T. D group. In the treated group, only intertubular hemorrhage and mild vacuolation in some tubules were observed. Furthermore, in the T. D group MSTD, GECT, TCT, and Johnson’s score were significantly reduced, and Cosentino’s score was increased. Leech therapy remarkably increased MSTD, GECT, and Johnson’s score. Furthermore, in the TDL group, Cosentino’s score was significantly lower than that in the T. D group. The results of the present study are consistent with previous findings. As shown in previous studies, saliva of leech consists of numerous ingredients that some of which act as vasodilators. These agents increase blood flow in the region of leech therapy and diminish inflammation [58]. Research on vasodilator agents has displayed an influential role of these agents in reducing the destructive effects of testicular ischemia/reperfusion [6]. Effects of sildenafil as a vasodilator was investigated on testicular torsion/detorsion, and it was concluded that the sildenafil treated group significantly increased MSTD, GECT, and Johnson’s score compared to the control group [59]. Trapidil, another vasodilator, was also assessed in the prevention of damages due to testicular torsion, and it could significantly increase MSTD, GECT, and MTBS [60].

In the present research, testicular torsion/detorsion significantly increased Bax and Caspase-3 positive cells and reduced Bcl-2 expression. Leech therapy reduced the percentage of positive cells to Bax and Caspase-3 and increased the Bcl-2 level indicating the reduction of apoptosis in the testis tissue. These findings are consistent with those of Ning and colleagues who found that testicular I/R significantly increased the Bax expression compared to the sham group and diminished Bcl-2 expression compared to the sham group. Treatment with MiR-29a reduced Bax expression and increased Bcl-2 expression [61]. Methylprednisolone and heparin protective effects on testicular torsion/detorsion were examined [55]. In the mentioned study, in the I/R group, the Bax and Caspase-3 IHC markers were significantly expressed. Bcl-2 was weakly expressed in the I/R group. Both methylprednisolone and heparin reduced Bax and Caspase-3 immunopositive cells and increased immunopositive cells to the Bcl-2.

## Conclusion

The main goal of the current study was to determine the protective effects of leech therapy on testicular ischemia/reperfusion in a rat model. This study has identified that leech therapy was effective in reducing oxidative damage, improving sperm quality parameters, decreasing histopathological damages, and reducing testicular apoptosis. These findings enhance our understanding of the role of leech therapy in reducing damage due to ischemia/reperfusion. The study is limited by the lack of information on the suitable time for leech therapy before detorsion. Further studies regarding the role of various times of leech therapy before testicular detorsion would be worthwhile to detect the best time for leeching.

## Data Availability

All data generated or analyzed during this study are included in this published article.

## Abbreviations

ROS: Reactive Oxygen Species
MDA: Malondialdehyde
GPx: Glutathione peroxidase
CAT: Catalase
TAC: Total antioxidant capacity
IHC: Immunohistochemistry
I/R: Ischemia/reperfusion
T.D: Torsion/detorsion
MSTD: Mean seminiferous tubular diameter
TCT: Testicular capsule thickness
GECT: Germinal epithelial cell thickness
